# Performance of Three LED-Based Fluorescence Microscopy Systems for Detection of Tuberculosis in Uganda

**DOI:** 10.1371/journal.pone.0015206

**Published:** 2010-12-28

**Authors:** Heidi Albert, Yukari Manabe, George Lukyamuzi, Patrick Ademun, Sheena Mukkada, Barnabas Nyesiga, Moses Joloba, C. N. Paramasivan, Mark D. Perkins

**Affiliations:** 1 Tuberculosis Research Laboratory, Foundation for Innovative New Diagnostics (FIND), Kampala, Uganda; 2 Infectious Diseases Institute (IDI), Mulago Hospital, Kampala, Uganda; 3 National Tuberculosis Reference Laboratory, Wandegeya, Kampala, Uganda; 4 Foundation for Innovative New Diagnostics (FIND), Geneva, Switzerland; Unité de Génétique Mycobactérienne, France

## Abstract

**Background:**

Direct smear microscopy using Ziehl-Neelsen (ZN) staining is the mainstay of tuberculosis (TB) diagnosis in most high burden countries, but is limited by low sensitivity in routine practice, particularly in high human immunodeficiency virus (HIV) prevalence settings.

**Methods:**

We compared the performance of three commercial light emitting diode (LED)-based microscopy systems (Primostar™ iLED, Lumin™ and AFTER®) for fluorescent detection of *Mycobacterium tuberculosis* with ZN microscopy on slides prepared from sputum of TB suspects. Examination time for LED-based fluorescent microscopy (LED FM) and ZN slides was also compared, and a qualitative user appraisal of the LED FM systems was carried out.

**Results:**

LED FM was between 5.6 and 9.4% more sensitive than ZN microscopy, although the difference was not statistically significant. There was no significant difference in the sensitivity or specificity of the three LED FM systems, although the specificity of Fraen AFTER was somewhat lower than the other LED FM methods. Examination time for LED FM was 2 and 4 times less than for ZN microscopy. LED FM was highly acceptable to Ugandan technologists, although differences in operational performance of the three systems were reported.

**Conclusions:**

LED FM compares favourably with ZN microscopy, with equivalent specificity and a modest increase in sensitivity. Screening of slides was substantially quicker using LED FM than ZN, and LED FM was rated highly by laboratory technologists. Available commercial systems have different operational characteristics which should be considered prior to programmatic implementation.

## Introduction

Direct (un-concentrated) smear microscopy using Ziehl-Neelsen staining is still the mainstay of tuberculosis (TB) diagnosis in most high burden countries, including Uganda, having remained essentially unchanged for over 100 years. This method is rapid and inexpensive and highly specific for *Mycobacterium tuberculosis* in high burden settings. However the main limitation of the method is its low sensitivity in programmatic settings, particularly in human immunodeficiency virus (HIV) co-infected patients [1]. High TB-HIV co-infection rates and consequent low TB case detection rates impede disease control in many TB endemic settings, notably in sub-Saharan Africa [2].

Auramine O fluorescence microscopy was first described by Hagemann in 1937 [3]. It has been estimated that fluorescence microscopy is approximately 10% more sensitive than Ziehl Neelsen (ZN) in detecting acid fast bacilli (AFB) in clinical specimens [1]. Furthermore, whilst the International Union Against Tuberculosis and Lung Disease (IUATLD) recommends at least 5 minutes of screening per slide to correctly identify a negative smear result [4], under routine field conditions, the time spent per slide is often far less than this recommended minimum time. Almost 50% of cases may be missed during routine slide examination [5].

However widespread implementation of fluorescent microscopy (FM) in disease endemic settings has not been realized. Primary reasons include high costs of equipment and mercury vapour lamps, short lamp lifespan (200–300 hours), the need for a stable power supply (as repeated on-off switching reduces lifespan of lamp), lack of local capacity for maintenance, need for a darkroom, and poor acceptance by laboratory staff.

Light emitting diodes (LEDs) for fluorescence microscopy have been recently introduced for screening of *Mycobacterium tuberculosis* [6], [7]. Several commercial LED systems are now available, either as stand-alone microscopes, or as add-on adapters to conventional microscopes [8]. LED-based fluorescence microscopy (LED FM) has several potential benefits compared with conventional fluorescence microscopy. LEDs provide a cheap and reliable light source with a long lifespan (>50 000 hours), repeated on-and-off switching does not reduce lifespan, and no darkroom is required for their operation. Replacement of light microscopy with fluorescence microscopy would be one of the immediate options for improving TB case detection in high-burden settings.

Data from reference laboratory settings have demonstrated that LED FM gives similar increases in performance and speed as FM using much more expensive conventional fluorescent microscopes [9] and is well accepted by end users [9], [10]. Large scale demonstration projects are being undertaken by the Foundation for Innovative New Diagnostics (FIND) in collaboration with National Tuberculosis Control Programmes in 10 countries to assess the performance of the Primostar iLED™ device in microscopy centres without previous experience with FM. Preliminary data from 9 microscopy centres in India reported greater than 95% agreement with conventional FM re-checking results within 1 month of implementation and equivalent or better accuracy compared with ZN microscopy within 2 months of use. End users rated the ease of use of LED FM as being greater than light microscopy [9], [11]. The World Health Organisation (WHO) has recently published policy recommendations on the use of LED FM in disease endemic settings, recommending that LED FM should replace conventional light microscopy in a phased manner [12].

This study sought to directly compare the performance of three commercial LED-based systems for fluorescence microscopy detection of *M. tuberculosis* with light microscopy in a research laboratory setting. Routine fluorescence microscopy performed at a hospital microbiology laboratory was also compared with all methods.

The three LED FM systems evaluated were (a) Primostar iLED™ (Carl Zeiss Microimaging, Oberkochen, Germany), a stand-alone microscope with reflected light source [13], (b) Lumin™ (LW Scientific, Lawrenceville, GA, USA), an LED objective adaptor using reflected light source [14], and (c) AFTER® (Amplified Fluorescence (by) Transmitted Excitation (of) Radiation) LED fluorescence add-on kit (Fraen SRL, Settimo, Italy), using transmitted light [15].

## Methods

Leftover portions of sputum specimens submitted by patients being investigated for pulmonary tuberculosis at Mulago Hospital complex were utilized in this study. Testing was performed between 27 January 2009 and 12 March 2009. Specimens were subjected initially to routine direct fluorescence microscopy in the Mulago Hospital Microbiology laboratory. Specimens received for follow up of treatment were excluded.

Up to a total of 30 specimens per day were selected (all samples if less than or equal to 30 samples were received, or the first 30 specimens). Specimens were transferred to a refrigerator upon receipt at the laboratory. Leftover portions were transported to the FIND Tuberculosis Research Laboratory situated at the National Tuberculosis Reference Laboratory, where all further testing was performed.

Two direct smears were prepared per specimen and stored in a slide box. Mycobacteria Growth Indicator Tube (MGIT) and Lowenstein-Jensen (LJ) culture were performed according to standard methods [16] with Capilia TB-Neo test (Tauns Laboratories, Inc.) used for *M. tuberculosis* identification [17]. Staining reagents for ZN and auramine staining were prepared according to standard procedures [18]. Positive and negative control slides were included in each batch.

Slide reading was performed by 2 technologists; reading of both smears from one specimen was performed by a single technologist (ZN plus all LED methods). An over-labeling (blinding) system was implemented by a study coordinator not involved in laboratory testing to avoid interpretation bias.

The study was approved by Makerere University and Mulago Hospital Research and Ethics Committee.

### Microscopy

The following LED FM systems were evaluated:

1. Primostar iLED (iLED) microscope
2. AFTER® (Fraen AFTER) adaptor, attached to Olympus CX31 microscope
3. Lumin™ adaptor, attached to Olympus CX31 microscope

Ziehl-Neelsen stained slides were read using the Primostar iLED microscope. The conventional fluorescence microscopy was performed using a NIKON Eclipse E200 microscope.

One auramine slide was read using all three LED FM methods. The order of reading was alternated with each batch to avoid bias due to possible fading of fluorescent stain with repeat reading. Slides were read 2 days apart on each LED FM system, and were not re-stained. Fluorescent smears were read at ×400 magnification with all methods. Grading of smears was according to WHO/IUATLD guidelines [18]. Grading charts were used for reading of all slides to allow quantitative comparison of the results using the different systems. 40 fields were read for fluorescence smears and 100 fields for ZN smears.

For quality assurance purposes, each reader examined a blinded panel of 30 slides each by ZN and by LED FM (10 slides by each method) prior to the start of reading smears from clinical specimens. Acceptable performance comprised no high false (HF) results, less than or equal to 3 low false (LF) results and less than or equal to 3 quantification errors (QEs) [19].

In addition, slides were randomly selected for re-reading for intra- and inter-reader variability using both ZN and LED FM methods throughout the study period. The readers were blinded to the previous results and the fact that the slides were for re-checking. In addition, any slides in which HF or LF results were obtained were re-read blindly by the other reader.

Routine fluorescence microscopy was performed at Mulago Hospital Microbiology Laboratory and was not subject to any intervention or quality assurance procedures by the study team.

### Culture and identification

After smear preparation, sputum was decontaminated by standard NALC-NaOH procedure (1.5% NaOH final concentration) [16]. Following neutralization and centrifugation the pellet was suspended in 1 ml phosphate buffer pH 6.8. 0.5 ml was used to inoculate MGIT culture and 0.1 ml each to inoculate 2 LJ slopes. Positive cultures were identified as *M. tuberculosis* using the Capilia TB-Neo assay.

### Examination time

A panel of 40 slides (20 per reader), a sub-set of slides prepared from the patients' specimens, was used for measurement of examination time using each method. A standardized form was used for data collection. An average examination time was calculated per result (negative, very low positive [scanty], low positive [1+] and high positive [2+ and 3+] for each method. The examination time included the time taken to record results.

### Data analysis

Standard statistical tests were performed using Intercooled STATA 8.0 software (Statacorp LP, College Station, TX, USA) and Microsoft Excel 7.0 (Microsoft Corporation, Redmond, WA). Results were considered significant at p<0.05. Sensitivity and specificity (95%CI) were calculated for each method compared with culture as gold standard. The sensitivity and specificity of the methods were compared in a pairwise fashion and McNemar's test for equality of proportions for paired samples was performed. The non-parametric Wilcoxon Rank Sum test was used to compare examination times (non-normal distribution).

## Results

### Panel slide results

Both readers passed panel slide reading for all methods on the first attempt. In total (combined results for Reader 1 and 2), 1 low false positive (LFP) was obtained for ZN, 0 errors were obtained for iLED, 4 LFPs and 1 low false negative (LFN) were obtained for Fraen AFTER and 1 LFP was obtained for Lumin. No QEs or HF results were obtained for any method.

### Performance of LED FM methods and ZN

A total of 193 specimens had results for microscopy, culture and species confirmation. A total of 53 specimens were culture positive for *M. tuberculosis*. 127 samples were culture-negative. Non-tuberculous mycobacteria (NTM) were isolated from 13 sputum specimens. Of these, 2/13 were smear positive by all LED FM methods, and 1/13 by ZN. These specimens were excluded from the analysis, leaving 180 specimens to be analysed.

Results of LED FM, ZN and routine FM are presented in Table 1. Sensitivity of the LED FM methods was between 5.6% and 9.4% higher than ZN. However, the difference was not significant at the 5% level for any of the methods (ZN vs Fraen AFTER, p = 0.063; ZN vs iLED, p = 0.125; ZN vs Lumin, p = 0.375). There was no significant difference in sensitivity when comparing the three LED methods with each other in a pairwise fashion.

**Table 1:** Sensitivity and specificity for detection of TB in slides prepared from sputum samples from TB suspects.

	ZN	Routine FM	iLED	Fraen AFTER	Lumin
**Sensitivity in culture positive sputa (%; 95% CI)**	33/53 (62.3%; 47.9–75.2)	29/53 (54.7%; 40.4–68.4)	37/53 (69.8%; 55.7–81.7)	38/53 (71.7%; 57.7–83.2)	36/53 (67.9%; 53.7–80.1)
Very low positive (scanty)	2	-	6	6	7
Low positive (1+)	8	-	9	10	8
High positive (2+, 3+)	23	-	22	22	21
**Specificity in culture negative sputa (%; 95% CI)**	125/127 (98.4%; 94.4–99.8)	127/127 (100%; 97.1–100.0)*	126/127 (99.2%; 95.7–100.0)	121/127 (95.3%; 90.0–98.2)	126/127 (99.2%; 95.7–100.0)

*one-sided, 97.5% confidence interval.

A different grading scheme was used for routine FM and hence grading results were excluded from analysis.

The specificity of Fraen AFTER was lower than the other methods. However the difference between methods was not significant at the 5% level. All false positive results were very low positive (scanty) results by all methods.

### Discrepant results

Any slides in which ZN and LED FM results did not agree were re-read by both methods in a blinded fashion shortly after initial reading. There were 2 false positive ZN results (1 and 3 AFBs observed), which were negative by all LED FM methods and negative upon re-reading ZN slide. One specimen was false-positive (18 AFB) on iLED, but was negative on re-reading and by all other methods. One slide was false-positive on both Lumin and Fraen AFTER (1 AFB observed on each), and a further 6 slides were false positive by Fraen AFTER only (with between 1 and 6 AFB observed per slide).

### Performance of routine FM compared with ZN and LED FM

Sensitivity of routine FM was the lowest of all methods, and was 7.6% less sensitive than ZN, although the difference was not significant (p = 0.388). Specificity of routine FM was equivalent to the other methods. A different grading scheme was used for routine FM and therefore the grading results were not directly comparable with the other methods and have been excluded.

### Difference in performance of 3 methods by different readers

There was a significant difference in sensitivity of microscopy achieved by the different readers: 40.9% and 77.4% for ZN (*p* = 0.007), 54.5% and 80.6% for iLED (*p* = 0.0415), 54.5% and 83.9% for Fraen (*p* = 0.019), and 50.0% and 80.6% for Lumin AFTER (*p* = 0.019), for Reader 1 and 2 respectively (Table 2). However there was no significant difference between readers in specificity for any of the methods. There was no difference in the ranking of sensitivity and specificity of the ZN and LED FM methods by the two readers.

**Table 2:** Per reader analysis of performance of ZN and LED-based fluorescence microscopy.

Reader 1				
	ZN	iLED	Fraen AFTER	Lumin
**Sensitivity in culture positive sputa (%; 95% CI)**	9/22 (40.9%; 20.7–63.6)	12/22 (54.5%; 33.2–75.6)	12/22 (54.5%; 33.2–75.6)	11/22 (50.0%; 28.2–71.8)
Very low positive (scanty)	1	2	2	2
Low positive (1+)	2	4	4	3
High positive (2+, 3+)	6	6	6	6
**Specificity in culture negative sputa (%; 95% CI)**	62/62 (100%; 94.2–100.0)*	61/62 (98.4%; 91.3–100.0)	59/62 (95.2%; 86.5–99.0)	62/62 (100%; 94.2–100.0)*

Reader 2				
	ZN	iLED	Fraen AFTER	Lumin
**Sensitivity in culture positive sputa (%; 95% CI)**	24/31 (77.4%; 58.9–90.4)	25/31 (80.6%; 62.5–92.5)	26/31 (83.9%; 66.3–94.5)	25/31 (80.6%; 62.5–92.5)
Very low positive (scanty)	1	4	4	5
Low positive (1+)	6	5	6	5
High positive (2+, 3+)	17	16	16	15
**Specificity in culture negative sputa (%; 95% CI)**	63/65 (96.9%; 89.3–99.6)	65/65 (100%; 94.4–100.0)*	62/65 (95.4%; 87.1–99.0)	64/65 (98.5%; 91.7–100.0)

*one-sided, 97.5% confidence interval.

### Intra and inter-reader variability

A total of 129 randomly selected slides were re-checked for intra-reader variability. Results of intra-reader and inter-reader variability for two readers are shown in Table 3.

**Table 3:** Intra and inter-reader variability.

	Reader 1		Reader 2	
	Intra-reader (n = 59)	Inter-reader (n = 70)	Intra-reader (n = 54)	Inter-reader (n = 72)
**ZN**	1 major error (HFP)	1 major error (HFP)	2 minor errors (1 LFP & 1 LFN)	1 minor error (LFP)
**iLED**	0 errors	5 minor errors (4 LFP & 1 LFN)	1 minor error (QE)	1 minor error (LFP)
**Fraen AFTER**	1 minor error (LFP)	5 minor errors (5 LFP)	0 errors	7 minor errors (6 LFP & 1 LFN)
**Lumin**	3 minor errors (1 LFN & 2 LFP)	1 major error (HFP) and 3 minor errors (1 LFP & 2 LFN)	0 errors	1 major (HFP) & 1 minor error (QE)
**Total**	**1 major & 4 minor errors**	**2 major errors & 14 minor errors**	**3 minor errors**	**1 major & 10 minor errors**

Intra-reader variability refers to re-reading of slides by the same reader.

Inter-reader variability refers to re-reading of slides by a second reader.

HFP, high false positive; LFP, low false positive; LFN, low false negative; QE, quantification error.

### Examination time

Average examination times for each method are shown in Table 4. Examination times for all LED FM methods were significantly shorter than ZN (p<0.001 for each pairwise comparison). Examination times for iLED and Fraen AFTER were similar equivalent, and were both significantly shorter than for the Lumin system (p = 0.0034 and p = 0.0138 respectively).

**Table 4:** Average Examination time of ZN and LED FM methods, related to smear grading.

Smear result	Examination time (mins), median (inter-quartile range)			
	ZN	iLED	Fraen AFTER	Lumin
**Negative**	5.08(4.51–6.07)	2.35(2.07–2.90)	2.29(1.97–3.09)	2.95(2.62–3.43)
Very low pos (scanty)	6.03 *	2.62(2.15–3.08)	2.50(2.40–3.05)	2.97(2.53–3.83)
Low pos (1+)	8.87*	2.80 *	3.84(2.55–5.12)	5.47 *
High pos (2+, 3+)	4.07(3.28–6.43)	1.04(0.74–3.69)	0.82(0.73–2.45)	1.25(0.73–1.30)
Overall	5.1(4.5–6.1)	2.3(2.0–2.9)	2.38(1.97–3.05)	2.94(2.49–3.47)

*1 slide only.

### End-user appraisal

A qualitative end-user analysis of the three LED FM systems was carried out after about 3 months experience with the LED FM methods. Responses were compiled and are presented in Table 5.

**Table 5:** User appraisal of LED FM systems.

	iLED	Fraen AFTER	Lumin
Installation	Easy	Difficult	Very easy
**Overall handling and features**	Superior to usual microscope	Inferior to usual microscope: add-on is bulky and inhibits slide placement on stage	Inferior to usual microscope: power cable of device interferes with stage movement
**Light intensity, contrast and background**	Homogeneity of illumination superior to usual microscope, adjustable light intensity	Light intensity too high; constant light intensity problematic for varying smear thickness	Light intensity too low and not adjustable, poor contrast
**Resolution and depth of focus**	Satisfactory	Satisfactory	Unsatisfactory
**Need for darkroom**	No	No	Partial – lights need to be switched off and windows covered
**Magnification**	×10, ×20, ×40 and ×100 objectives (for ZN and FM)	Magnification depends on base microscope.	Device attaches to single objective, magnification chosen when purchasing (×20, ×40, ×60, ×100 available)
**Use of ZN and FM on same system**	Easy to switch between ZN and FM modes	Difficult to add and remove device, would not use same microscope for LED FM and ZN on same day	Easy to add and remove - possible to use for ZN and LED FM on same day
**Power supply**	Battery pack available	Battery pack available	No battery pack

## Discussion

The objective of this study was to give a direct comparison of three LED-based systems for fluorescence microscopy for TB detection with ZN microscopy, the current standard used in disease endemic settings. Furthermore, the study also allowed comparison of LED FM and ZN microscopy performed in a research laboratory setting with routine conventional FM performed in a busy hospital microbiology laboratory.

Sensitivity of LED FM was between 5.6% and 9.4% higher than ZN, although the difference was not significant for any of the methods due to the small sample size in this study. This is similar to findings of a recent systematic review of 45 studies in which conventional fluorescence microscopy was on average 10% more sensitive than conventional light microscopy [1].

Routine FM performance in this study was lower than all other methods (although not statistically significant), including ZN, pointing to issues relating to quality of performance of the routine FM. Factors such as quality of smear preparation, staining and length of time spent reading slides may have contributed to the low sensitivity of the routine FM. The readers performing routine FM were outside the study team and hence were not subject to the quality assurance measures in place for the study. These operator-dependent factors remain critical to performing high quality microscopy, irrespective of the system used.

Specificity of the LED FM was not significantly different than the specificity of ZN microscopy. This agrees with reports for specificity of conventional FM in which specificity of FM and ZN were found to be similar [1].

Examination of slides was 2 to 4 times faster for LED FM than ZN. It is likely that these results underestimate the time-saving benefit of FM since the use of grading charts and the requirement for accurate quantification of low-positive results slowed examination in the study. Nonetheless, a substantial reduction in examination time was demonstrated. A previous study comparing fluorescence and conventional microscopy found that higher sensitivity and equivalent specificity were achieved with 1 minute examination of FM slides compared with 4 minutes examination of ZN slides [20].

A difference in performance of the two readers was observed. Reader 2 was more experienced in microscopy and had performed fluorescence microscopy prior to starting the study, whereas Reader 1 had previously performed ZN microscopy only. However both readers passed the initial proficiency panel on the first occasion, and had similar performance in intra and inter-reader variability (Reader 2 had slightly lower rate of errors). Nevertheless, since the difference in sensitivity of reading was observed for all methods and the ranking of sensitivity of the three LED FM methods was the same for each reader, it is unlikely to have caused significant bias in the overall analysis. This finding does support the need for very close monitoring of readers in the early stages of implementation of fluorescence microscopy. Furthermore, it should be noted that this variability in the performance of the two readers may have led to an over- or under-estimation of the sensitivity improvement achieved using LED FM.

User acceptance of a new technology is critical in its successful uptake and widespread implementation. Indeed, poor user acceptance has been given as one of the reasons for lack of implementation of conventional fluorescence microscopy [10].

This study, the first to directly compare three commercial LED FM systems head to head, found several differences in operational characteristics of the three systems which may impact user acceptability. Firstly, the iLED microscope has adjustable light intensity which was found to be desirable especially when examining slides with varying smear thickness. The other two systems have fixed light intensity, which was considered suboptimal. Secondly, the availability of different objectives for use with LED FM was considered advantageous, and was available for iLED (a stand-alone microscope) and for Fraen AFTER. The Lumin adaptor attaches to a single objective, and therefore a single magnification must be selected at the time of purchase. Both LED adaptors inhibited the ease of use of the Olympus ×31 microscope used in this study, due to limitations in placement of the slide and stage movement. It is not known whether this problem is specific to this microscope or a more general design issue. Lastly, we considered that due to the complexity of installing the Fraen AFTER add-on, it was not feasible to use the same microscope routinely for light microscopy and LED FM on the same day. The Lumin system was simple to install, and the iLED switches instantly from bright light to LED FM, allowing both to be used for both applications in a single working day. However, overall all LED FM systems evaluated were considered a favourable alternative for screening of sputum smears for TB detection compared with conventional light microscopy. Affolabi *et al.* [21] recently reported results of a two-way comparison of the Fraen and Lumin systems, in which the two devices performed similarly at ×400 magnification, but Fraen performed better than Lumin device at the lower magnification (x200). In our study we looked at a single magnification (x400) for reading with all three methods, and did not study performance of the LED devices at different magnifications.

Other commercial LED FM systems are also now available [8], both stand-alone instruments and add-on kits, as well as other systems in development [22], [23].

Minion *et al* reviewed costs of LED FM systems in 2009, reporting prices between $700 and $3530 for LED adaptors, depending on sales volume and model, and $4825 for the Primostar iLED [8]. Since the WHO approval of LED FM for use in low and middle income countries in 2010 [12], the price of LED microscopes has reduced significantly in disease endemic countries. For example, the current FIND-negotiated price for the Primostar iLED microscope is €1250 (approximately $1750) for low and middle income countries [24].

A secondary finding of this study was that performance of ZN microscopy under ideal conditions can be equivalent to routine FM. This was most likely due to issues in smear preparation, staining quality and/or length of time spent reading the smears. This highlights the necessity of emphasising the basic components of quality microscopy when implementing a new technology improvement, since the overall quality of results is highly dependent on a number of operator-related factors.

This study was carried out in a research laboratory setting. A larger implementation study will investigate the operational performance of the three LED FM systems in screening 810 TB suspects presenting at an HIV clinic at the Infectious Disease Institute, Mulago Hospital, Kampala. These data will add to the accumulating body of evidence on the successful implementation of LED FM in peripheral settings in disease endemic countries [10], [11], [25], as well as providing a sufficiently powered sample size for comparison of operational performance of the methods in a cohort of HIV-positive TB suspects.

In conclusion, we demonstrated the improved performance of three LED FM systems compared with conventional light microscopy in a research laboratory setting. The size of the study did not allow demonstration of significant differences in performance between the three LED FM methods used except for a slightly lower specificity found with the system using transmitted light. Additional data from larger scale studies would be needed to delineate differences in detection performance of the various LED systems now commercially available. Significant differences in operational features of the LED FM systems were observed which should be considered prior to programmatic implementation. Furthermore the minimum training requirements for laboratory staff without prior FM experience should be further investigated and close monitoring of LED FM performance post-implementation should be prioritised to ensure the full potential benefits of the technology can be gained in routine practice.
